# Medical News and Miscellany

**Published:** 1887-01

**Authors:** 


### MEDICAL NEWS AND MISCELLANY.

---

DR. T. J. TYNER, late of San Antonio, has removed to Austin.

---

DR. J. R. BRIGGS will reply to Dr. Wallace's criticism of his Prize Essay, in our next issue.

---

DR. A. WADGYMAR, at Corriz Springs, Texas, wants to sell his practice. Address him as above.

---

Dr. B. E. HADRA, of Austin, has gone to Chihuahua, Mexico, to perform some gynaæcological operations by request.

---

MARRIED in Georgetown, Texas, January 7, 1887, Dr. W. T. Jones to Miss Allie M. Snyder, daughter of Col. D. H. Snyder all of that city.

---

Cincho Quinine does not produce that disagreeable, nervous irritation, so dreaded by many sensitive persons, which we call "Quinism."

---

THE NAME OF THE JOURNAL OF CUTANEOUS AND VENEREAL DISEASES has, very properly, been changed to the Journal of Cutaneous and Genito-Urinary Diseases.

BILLS FOR VOL. II, (July, '86, to June, '87), will be sent this month to all whose subscriptions have expired, and prompt payment is respectfully requested.

---

MARRIED at St. David's church, in Austin, January 5th, by Rev. T. B. Lee, Dr. Ralph Steiner to Miss Lilly Bremond, daughter of Eugene Bremond, Esq., all of this city.

---

DIED.—In Ennis, Texas, November 11th, 1886, Dr. J. T. Meek, of that city. Dr. Meek was an active member of the Ellis County Medical Association and of the Texas State Medical Association.

---

Report of State Health Officer of Texas and Report of Superintendent of the State Lunatic Asylums (at Austin and at Terrell) also of Superintendent of Institute for the Blind, have been received.

---

PERSONAL.

Doctor Fred K. Fisher, late health officer at Pass Cavallo, who lost his all in the August storm, has removed to Galveston to practice his profession.

---

THE GALVESTON MEDICAL CLUB will give their annual dinner at the Tremont Hotel, Monday, January 26. We acknowledge the courtesy of an invitation, and regret it is impossible for us to be present.

---

WE REGRET TO NOTE the retirement of Dr. Robert Leudeking from the associate editorship of that live and interesting weekly, the St. Louis Weekly Review. He is succeeded by Dr. B. J. Primm, and Dr. Y. H. Bond assumes charge of the Department of Gynæcology.

---

A BIG MEDICAL BILL.—It is stated in the daily papers that Dr. R. H. Harrison, the Chief Surgeon of the Southern Pacific Railroad system in Texas is suing, in the courts at Houston for the recovery of $14,000, from the estate of the late President Pierce of the G.H.&S. A. Road, (which estate is valued at eight million dollars) for professional services claimed to have been rendered Mr. Pierce, during his last illness. One items is $5,000 for a visit to Massachusetts, where the Doctor was summoned in consultation with eminent local physicans.

THE NEUROLOGICAL REVIEW.—We regret to learn that, in consequence of continued ill health, our esteemed colleague, Dr. J. S. Jewell, has been obliged to suspend publication of the Review. He hopes to resume, if his health will permit, after a year's rest.

---

THE ST. LOUIS POST-GRADUATE SCHOOL OF MEDICINE, in view of the low charges for medical teaching at other schools, has reduced the terms to thirty dollars for the course of six weeks, including all departments. The clinical facilities of this Institution are unrivalled in variety and instructiveness. For information, address P. G. Robinson, M. D., Dean, or Charles E. Briggs, M. D., Secretary, Polyclinic, S. W. Cor. Lucas & Jefferson Aves.

---

REPORT OF THE BOARD OF REGENTS OF THE TEXAS UNIVERSITY.—We acknowledge the receipt of a copy of this interesting report, at the hands of the President, Dr. T. D. Wooten. We are glad to see he touches upon the important point of a Medical Department, and of the necessity of its being at the capital—the seat of the University. In our next we hope to have something to say on the subject, and to second the distinguished President in his views.

---

ANOTHER NEW JOURNAL, THE SOUTH WESTERN MEDICAL JOURNAL.—Somersville, Kentucky, a monthly Journal of Medicine and Surgery, edited by M. F. Coombs, A. M., M. D. and J. B. Marvin, B. S. M. D. $1.00 a year in advance. We have read Vol. 1, No. 1. It carries on title page a cut of two of Kentucky's most renowned Surgeons, McDowell and Du dley. Sailing under such colors the standard of excellence should be kept high, as doubtless it will be. Success.

---

TRANSACTIONS OF THE TEXAS MEDICAL ASSOCIATION.—Eighteenth Annual Session, held at Dallas, Texas, April, 1886.

This is a ponderous volume of nearly 700 pages, handsomely bound and printed. It is one of the most complete volumes of the kind that we have seen, and in the appearance of its transactions and the fulness of the reports, the Texas State Medical Society holds an easy lead. Much of the credit for such a beautiful volume is undoubtedly due to Dr. F. E. Daniel, the secretary of the society, and the editor and publisher of *Daniel's Medical Journal.—Independent Practitioner.*

PRACTICE FOR SALE.

In Northern Mexico, at a flourishing railroad town. A practice without competition; worth $2,500.00 a year in cash. Can be bought on reasonable terms. Address, C. K. G., care Daniel's Medical Journal, Austin, Texas.

---

HYPERBOLE.—An esteemed Texan exchange thus anticipates 1887: "We may feebly, therefore, forecast the policy of the ——— for the coming year. Brimful of energy, enterprise, and financial backing, the ——— will start, with an invigorated and undaunted spirit, on the future labors which are horoscoped in the new year that will soon dehisce, as it were, into the full glory of an actual, living reality."

If the above is "feebly" expressed, what would have happened had the editor been feeling first-rate?—*Columbus Medical Journal.*

The Columbus Medical Journal is referred to the "Prize Essay," as a specimen of what the Doctor can do when he turns himself loose.

---

"THERE'S MUSIC IN THE AIR."—The plot thickens. In our last issue appeared a criticism by Dr. D. R. Wallace, of Terrell, Texas,on Dr. Briggs' "Prize Essay." In the closing paragraph Dr. Wallace challanges Dr. Briggs to submit his paper to three well-known and recognized psychologists, of national reputation, one to be selected by Dr. Briggs, one by the President T. S. M. A. and one by Dr. Wallace, and if they decide that the Essay is a prize paper, he Dr. Wallace agrees to pay Dr. Briggs an additional $100; if not, Dr. Briggs shall return to the treasury the $100 awarded him by the State Association. Dr. Briggs has acepted the challenge and offers to put up $50 forfeit, and demands that Dr. W. shall do the same. Dr. Briggs' reply will appear in an our next issue.

---

GAILLARD'S MEDICAL JOURNAL. — This sterling publication comes to us for January replete with valuable, interesting matter, both original and selected. It shows, in its management, a consummate ability, and a thorough appreciation of the wants and tastes of the reading portion of the medical public. Dr. Porter, the accomplished editor who has so signally assisted Mrs. Gaillard—who is daughter of one of Virginia's most distinguished physicians,—in the commendable determination to keep the Journal up to that high standard of excellence which the lamented

Gaillard gave it, seems to possess a rare and happy faculty of selection for the benefit of his readers, as well as a fruitful source whence to obtain that which is new and valuable. The ranks of medicine in Texas are adorned by numerous pupils of the distinguisted Prof. Gaillard, graduates of the school to which he lent the lustre of his name and the wealth of his knowledge, and they who love his memory, seeing the effort in their interest, which is being made by his accomplished widow,should rally to her assistance with purse and pen; it is a duty they owe, no less to his memory than to her devotion and worthy endeavors. Her struggles and successes are without a parallel,—left, as she was, with a family of young children, and totally unqualified, by birth,education and position for a struggle with the world, and with no heritage other than the JOURNAL and the fame of its illustrious founder. All honor to the brave daughter of Virginia who so nobly seized the helm, warm from the hand of her gallant husband, and steered it through the straits of adversity, to a safe haven. May she never want for friends to love her for her worth, and admire her for her courage.

---

THE NEW ADMINISTRATION AND THE STATE HEALTH OFFICE—OTHER MATTERS.—Governor Ross has signalized the beginning of his administration by an apparent disregard of the wishes of the people in the matter of appointment of State Health Officer,—and like Cæsar he turns a deaf ear to the petition of his Senators. Dr. Swearingen, during his three terms of office, has given evidence of the highest order of ability, and by his wise administration, by which Texas has been spared the ravages of epidemic diseases when more than once threatened,—no less than by his admirable social and professional qualities, has endeared himself to the people, and they desire and have petitioned for his reappointment. But, it seems, neither eminent and successful service—extending over a period of six year—and pre-eminent fitness for the discharge of the duties of this responsible office, nor the wishes of the people unmistakably expressed, will avail. It would seem that the nature of the office, and the magnitude of interest involved should put it beyond the realm of mere political influence; yet Governor Ross without the shadow of a cause, and in the face of his most brilliant record, and despite the clamor for his reappointment, has indicated a wish to supplant him, by the appointment of another. The Senate with the exception of three members, petitioned for Dr. Swearingen's reappointment; the entire profession at Galveston, the most important sea-port city, have urged it; numerous petitions extensively signed, and by all classes of people, have poured in, but the Governor last week sent in the name of Dr. Rutherford, of Houston, for the place. Dr. Rutherford would doubtless make an admirable health officer, and would be a worthy successor to the present incumbent, were it necessary or desirable to make a change; but it seems to be the almost universal wish of the people, so far as we can learn, that Dr. S. be continued in office. The Senate up to the present writing (Jan. 24) have taken no action on the subject, but have postponed consideration of it four days,—meantime public sentiment is divided as to whether or not the appointment will be confirmed.

Governor Ross' first official act in this department, was to name Dr. J. S. Dorsett, of Bonham, for successor to Dr. A. N. Denton, Superintendent of the State Lunatic Asylum. The Board of Directors refusing to confirm the appointment, not because there was objection to Dr. Dorsett but because in their judgement Dr. Denton should be retained, the Governor dissolved the Board, and appointed one which, it is understood, will respect his wishes, and it is considered settled that Dr. Dorsett will relieve Dr. Denton, February 1st. Meantime Dr. Dorsett will make no changes in the medical staff. It is rumored that Dr. Wallace at Terrell will be removed also. Hon. G. W. Kendall of Denton county, is to relieve Dr. Shepard in charge of the Deaf Mute Asylum, and Dr. Frank Rainey of the Blind school is the only superintendent whose official head is not to fall under the political axe.

During inauguration week many distinguished physicians visited the Capital, some office seeking, many sight seeing. Amongst the number, we were pleased to meet Drs. Pope, Smith and Welch, of Dallas; Dr. Jordan of Beaumont; Dr. Talley, of Belton; Dr. Burleson, of Weberville; Dr. Shropshire, of Brownwood; Dr. Wallace, of Terrell; Dr. Rutherford, of Houston, and Dr. G. W. Christian, of Burnett. Apropos; Dr. Christian has been appointed Surgeon (Major) of Second Regiment Texas Volunteers, and in his regimentals looked "every inch a soldier."

